# Mobile Helical Capacitive Sensor for the Dynamic Identification of Obstructions in the Distribution of Solid Mineral Fertilizers

**DOI:** 10.3390/s18113991

**Published:** 2018-11-16

**Authors:** Ricardo Dalacort, Sergio Luiz Stevan

**Affiliations:** 1 Graduate Program in Applied Computing (PPGCA), State University of Ponta Grossa (UEPG); Ponta Grossa (PR) 84030-900, Brazil; ricardo_dalacort@hotmail.com; 2 Graduate Program in Electrical Engineering (PPGEE), Federal University of Technology of Parana (UTFPR), Ponta Grossa (PR) 84016-210, Brazil

**Keywords:** capacitive sensor, dielectric permittivity, fertilizer distribution, dosing mechanism

## Abstract

Modern agriculture uses techniques and technologies that have provided farmers with increased yield and a possible reduction in costs. Optimizing the use of inputs by applying exact and accurate doses, which match the real needs of the soil, in addition to supplying the necessary nutrients for the correct development of the crops, enables a reduction in costs and environmental impacts caused by the incorrect use of products such as fertilizers and pesticides. With this background, this paper presents a study on the development of a capacitive sensor to identify the absence, presence or variations in the distribution of solid mineral fertilizers. To evaluate this sensor, eight different formulations were tested in distribution analysis with an overflow dosing mechanism, both statically and dynamically, with 2% maximum moisture variation between all samples. The identification of an absence or presence of fertilizers was successful in 100% of the experiments. Tests to identify variations in the fertilizer distribution were carried out through simulated obstruction. The sensor identified a reduction in the fertilizer flow in all experiments, obtaining numeric variations above 55%. In the fertilizer formulation identification test, only the formulations 02-28-20 and 06-21-12 in experiments carried out with the overflow dosing mechanism did not differ statistically one from another, while all other formulations presented a statistically significant difference in the ANOVA analysis and the Tukey test at 5% significance.

## 1. Introduction

The use of scientific and technological knowledge to improve yield and for the possible reduction of costs is an important characteristic of modern agriculture, which might generate better economic results for farmers. An increase of the yield efficiency of a system mainly depends on the technology used with it, and its profitable use depends on the amount of information available to better use its resources. Within this perspective, precision agriculture (PA) appears to be an ally to the farmers, introducing techniques and technologies to help with the management and improvement of the agricultural production system. One of its main objectives is the local and specific management of the soil with the application of inputs in the right place, at the right time and in accurate amounts [1,2]. Thus, the amount of input is reduced, minimizing problems caused by irregular distribution and contributing to an increase in productivity [3,4].

Plant nutritional needs are determined by the amount of nutrients they receive during their development cycle. Therefore, the soil fertilization process is vital to the crop development, allowing the reposition of nutrients extracted by the plants or the addition of nutrients missing in that soil, providing greater sustainability to the farm production system [5]. However, this process is often carried out in an unsuitable way, harming the development of cultivars, reducing yield and contributing to environmental pollution [6].

**Figure 1:**
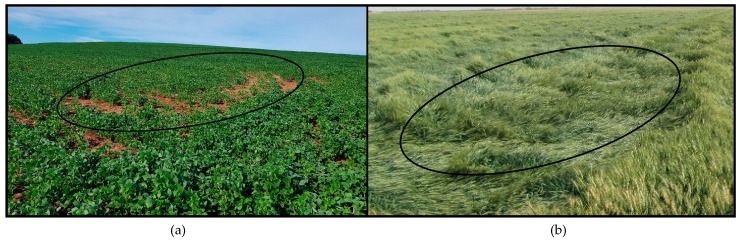
Problems caused by irregularity in the fertilizer distribution. (**a**) Fertilizer underdosing; (**b**) fertilizer overdosing.

The use of fertilizers, above or below requirements, results in crop development problems. The underestimation of the fertilizer results in a lack of the nutrients necessary for the development of the crop, producing yellowish leaves, while overdosing leads to soil acidification, problems related to phytotoxicity, plant accommodation and contributes to environmental degradation [7,8]. One of the factors favoring plant bedding is the high rate of nitrogen fertilization applied to cultivars that do not have the root or caulinar system needed to support this growth, resulting in curved plants and causing productivity losses [9,10,11]. Figure 1 illustrates these two situations, where there were subdosages applied to soybean (Figure 1a) and overdosages, which were more visible, on wheat crops (Figure 1b). Together with other factors such as a greater or lesser need for soil correction, characteristics of relief and characteristics of periodic rainfall, we can say that sub or overdosage of fertilizers contributes strongly to problems such as those exemplified. 

A regular distribution of fertilizers depends mainly on the quality of use of dosing mechanisms; however, these mechanisms are not accurate and do not present uniformity of distribution due to external factors such as inclination during the work caused by the soil relief, planting speed and the type of fertilizer used [12]. The study developed by [2] evaluated uniformity in the distribution of fertilizers by a pneumatic dosing mechanism at different speeds and application rates, and the evaluation criteria was the variation coefficient (VC). All the experiments carried out presented variations in fertilizer application; however, the best result was obtained with a 26 ${\mathrm{ms}}^{-1}$ speed at a 20 ${\mathrm{kg}\;\mathrm{ha}}^{-1}$ rate, presenting the lowest variation coefficient. Variations in fertilizer dosage carried out by a helical dosing mechanism were also reported by [13,14,15] when the mechanism was submitted to different longitudinal inclinations.

However, the uniformity in the distribution does not depend only on the machine characteristics or the adjustments employed; it also depends on the fertilizer’s physical characteristics such as granule size, hygroscopicity, fluidity and consistency, among others [16,17,18]. Variations in fertilizer distribution might be caused by obstructions resulting from the fertilizer aggregation, which generates particles that are larger than the original ones and might reduce the normal flow of distribution [19]. 

Some research has introduced technologies applied to the process of distribution of fertilizers seeking better efficiency and homogeneity in the distribution. Studies reporting different techniques and technologies used to quantify and monitor the fertilizer output flow in different dosing mechanisms are presented below.

A system to control fertilizer distribution at a variable rate was developed using sensors to monitor the fertilizer flow in real time. For this purpose, the fertilizer reservoir was substituted by a cylinder, keeping the level of the fertilizer in the reservoir uniform, thus enabling the verification of the depth of the fertilizer inside the cylinder by using an encoder coupled to the top of the cylinder, and consequently the amount of fertilizer deposited in the soil [20]. Another project proposed a dynamic system using weight sensors to determine the fertilizer output flow in a pneumatic dosing mechanism [21]. The study reported in [22], presented the construction and simulation of a system based on fuzzy logic to control the mass flow applied by a fertilizer dosing mechanism of the helical screw type. A prototype of an optical sensor was presented for the measurement of the mass flow of granular fertilizers released by helical dosing mechanisms in real time [23]. Also using optical sensors, [24] reported the development of an intelligent system to monitor and measure the flow of fertilizers and seeds, enabling through sound and light alarms the prevention of distribution irregularities caused by obstructions of output ducts or other similar problems. Another technique used is the alteration of capacitance, described in [25], where a capacitive sensor was developed containing three parallel plates inserted in the output duct connected to the dosing mechanism to measure the mass flow or fertilizers destined to the soil. 

Parallel to this scenario, there is still a constant distribution of fertilizers which are mechanically adjusted through streams, which can be interrupted by blockage/obstruction or by the completion of the contents of the reservoir. The fertilizer presence or absence control is usually carried out manually by the machine operator. The operator turns off the traction vehicle and verifies the fertilizer reservoir level; this procedure tends to reduce the efficiency and speed of the planting process. For many generations, an employee positioned on the planter has done the monitoring of fertilizer reservoir levels visually, but in addition to an increase in costs, this generates risk of work-related accidents. The norms for ensuring the safety of workers during the planting process are different in each country. In Brazil, the Regulating Norm 31 (NR-31), session 31.12.4, prohibits the transportation of people on automotive machines and implements. Thus, the automatic identification of the presence, absence or variations in the fertilizer distribution speeds up the planting process, making it more efficient and safer, preventing accidents due to violations of NR-31.

With this background, the need for constant research on sensors that provide improvements and a better control of the fertilizer distribution process becomes evident. Thus, this paper presents the development of a capacitive sensor, based on the helical fertilizer distribution system. From this proposal, the paper shows that it is possible to monitor the fertilizer dosing flow; that is, to dynamically identify the presence, absence or significant variations of dosage. Mainly, the results will demonstrate the proposed sensor’s ability to identify partial or total obstructions to the pre-set normal fertilizer application flow.

## 2. Materials and Methods

### 2.1. Capacitive Sensing

Capacitance refers to the ability to store electrical energy between the electrodes that make up a capacitor or capacitive sensor when they are submitted to potential difference. It depends on some factors, such as the electrode geometry, which is inversely proportional to the distance between electrodes, proportional to the common area of electrodes and the dielectric permittivity of the dielectric present between them, and as a function of these characteristics, it can be either analytically or numerically calculated [26]. 

The electric field in a certain dielectric medium is always generated perpendicular to the surface of the electrodes that involve it. It is practically uniform when the electrodes are parallel surfaces, except for the edges, where the fringe effect appears, which might have up to a 35% effect on the capacitance [27]. When the electrode geometry is not parallel, the field is not uniform, becoming stronger in the region where the electrodes are closer and weaker in the regions where they are more distant.

Capacitive sensors are largely used in industry and agriculture and are mainly employed to identify presence [28], level [29] or pressure [30], and usually use well defined characteristics such as geometry (flat or cylindrical) or face (frontal presence), which is completely different to that proposed in this study.

### 2.2. Development of a Mobile Helical Capacitive Sensor

The sensor developed is based on theoretical concepts related to alteration in the capacitance caused by the disturbance of an electric field, generated by two conducing electrodes charged with opposite electrical loads. Such disturbance is caused by the alteration of the amount of dielectric material present between the electrodes. The capacitive sensor developed is made up of two electrodes—one is semi-cylindrical and the other is helical—placed inside the dosing mechanism, and the helical electrode is positioned above the semi-cylindrical electrode.

The size of the electrodes directly influences the electric energy storage capacity of the capacitive sensor. The helical electrode is 104 mm long, with four complete loops separated by a 25 mm distance and 54 mm diameter. The semi-cylindrical electrode is 110 mm long with 60 mm diameter and was developed with stainless material in order to prevent possible oxidation caused by the fertilizer. The electrodes were separated by around 3 mm distance and placed in such a way that the fertilizer was placed between the electrodes, acting as the capacitive sensor dielectrics. 

Aiming at eliminating any possible effect of the electric conduction through the dielectrics, an acrylic resin layer was applied on the helical electrode. This acrylic resin layer along with the fertilizer and the air between the granules acted as the capacitive sensor dielectrics.

**Figure 2:**
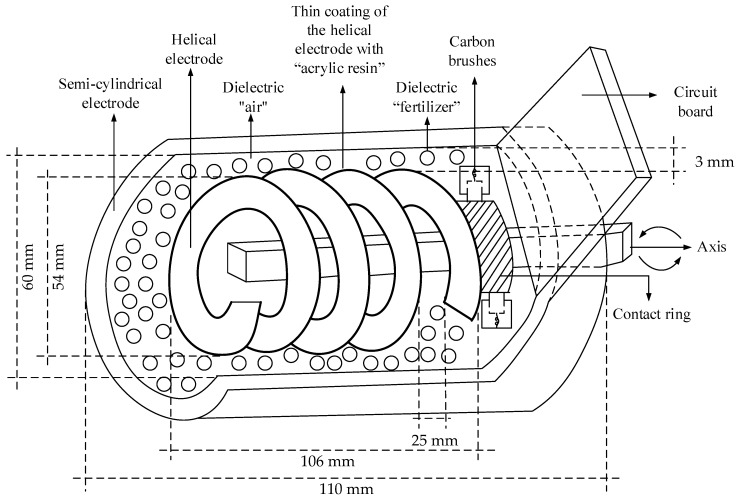
Developed sensor.

The helical electrode, in addition to being part of the capacitance sensor, is responsible for the transportation of the fertilizer to the output duct; that is, the sensor proposed can be considered a mobile helical capacitive sensor. Due to the need to energize the moving electrode, the helicoid was adapted by fixing a metallic ring to one of its ends, enabling feeding through carbon brushes. From the axis movement through the engine, the brushes remain fixed, “sliding” on the surface of the metallic ring and enabling the constant energization of the helicoid. This technique is commonly used inside engines and other machines that need to be energized while moving. Figure 2 illustrates the developed sensor with its respective dimensions and components needed for the energization in the dynamic mode.

#### 2.2.1. Analytical Approximation of the Theoretical Capacitance of the Developed Sensor 

Due to the geometrical irregularity of the electrodes that make up the sensor, the theoretical capacitance was approximated using the expression that corresponds to the capacitance of a parallel plate capacitor as the basis. For such approximation, both electrodes were made flat and analyzed in parallel.

The first point analyzed to carry out the analytical approximation of the sensor’s theoretical capacitance was the different intensities of the electric field due to the helical electrode height, since the electric field presents higher intensity where the distance between electrodes is smaller.

**Figure 3:**
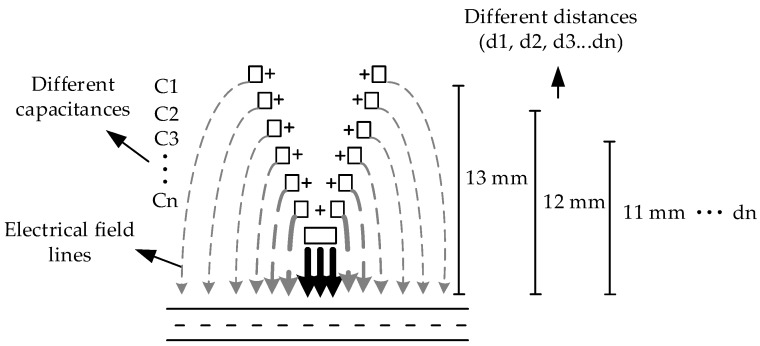
Illustration of the electric field different distances and respective capacitances, to front view of the flattened helical electrode.

The helical electrode was around 10 mm high, and the intensity of the electric field in the upper end is lower when compared to that of the lower end, resulting in different capacitances. The capacitance is known to be inversely proportional to the distance between electrodes. Thus, for a better approximation of the capacitance, the two sides of the helical electrode were “sliced”, obtaining equal areas; however, with different distances between the electrodes. Figure 3 illustrates the area and its respective distances along with the different capacitances due to the geometrical shape of the electrodes. 

The second point analyzed was the presence of different dielectrics inside the sensor, which were fertilizer, air and the (electrode coating) acrylic resin. Thus, based on the classical equation of parallel plate capacitors, we perform an analytical approximation of the theoretical capacitance of the sensor, considering it as a set of small associated elements. In this way, we take into account both the difference of distances and the contribution of the different dielectrics present between the electrodes, which are expressed through Equation (1).

(1) $$C=\overset{n}{\underset{j=1}{\sum }}\left[\varepsilon_{0}\cdot \left(\varepsilon_{r1}\cdot \left(\frac{\rho_{ap}}{\rho_{real}}-\frac{\alpha }{100}\right)+\frac{\varepsilon_{r2}\cdot \left(\rho_{real}-\rho_{ap}\right)}{\rho_{real}}+\frac{\alpha \cdot \varepsilon_{r3}}{100}\right)\cdot \left(\frac{A_{j}}{d_{j}}\right)\right]\cdot K$$

where $\varepsilon_{0}$ is a vacuum permittivity constant; A is the electrode area $\left(m^{2}\right)$; d is the distance between electrodes (m); $\varepsilon_{r1}$ is the fertilizer dielectric constant; $\varepsilon_{r2}$ is the air dielectric constant; $\varepsilon_{r3}$ is the acrylic resin dielectric constant; $\rho_{ap}$ is the fertilizer apparent density; $\rho_{real}$ is the fertilizer real density; α is the acrylic resin thickness; and K is the adjustment constant empirically determined as a function of mechanical inaccuracies, measurement errors, as well as simplifications and numerical approximations. 

It is important to note that the dielectric material between the electrodes is composed of granulated material, and that there is an amount of air filling the spaces. The proportion of air and fertilizer material is determined by the apparent densities that must be estimated experimentally according to the type/batch of fertilizer analyzed.

#### 2.2.2. Data Acquisition Circuit

Different studies [31,32,33] presented methods that enable capacitance measurement. From these, the widely used methods to measure outstanding capacitance are capacitive impedance [34], capacitance bridges [35] and oscillators [36]. 

In this study, a capacitance to frequency method was used to measure the capacitance. The integrated circuit LMC555 in its industrial version was employed, which is widely used in electronics due to its low cost and high versatility. This integrated circuit can be configured as a monostable multivibrator or as an astable multivibrator. In its astable configuration, it acts as an oscillator, producing a digital output with a certain frequency, which depends on two resistors and a capacitor connected externally to the circuit [36]. 

For the astable configuration, Equation (2) is used to calculate the capacitive sensor capacitance, once the value of the resistors (R1 and R2) and the frequency of the operation (f) have been defined.

(2) $$Cx=\frac{1.44}{\left(R1+2\cdot R2\right)\cdot f}$$

A rapid prototyping board (Tiva C Series TM4C1294) based on the Texas Instruments MCUTM4C1294NCPDT microcontroller (32 bits ARM Cortex^®^-M4F) was used to capture and process the output signal of the astable multivibrator circuit. The processed signal was transmitted serially to a computer so that the data could be collected and then analyzed.

### 2.3. Methodology of the Dynamic Evaluation/Control of Fertilizer Distribution Speed

The planter dislocation speed while sowing is determined by considering factors such as the crop implemented and the seed and fertilizer dosing mechanism, soil relief inclination, among others. Aiming at carrying out laboratory experiments, so that the speeds normally applied were respected, an engine was adapted to the axis of the dosing mechanism and a potency circuit was developed to control the speed.

An optical sensor was adapted to the axis of the dosing mechanism to count the axis rotations per minute (RPM) and adjust the axis rotation to simulate a distribution speed coincident to that used on the field. A microcontroller *MSP430* by Texas Instruments captures the axis in RPM, and through a firmware that generates a pulse-width modulation (PWM) signal, adjusts the tension applied to the potency circuit that alters the distribution speed. 

**Figure 4:**
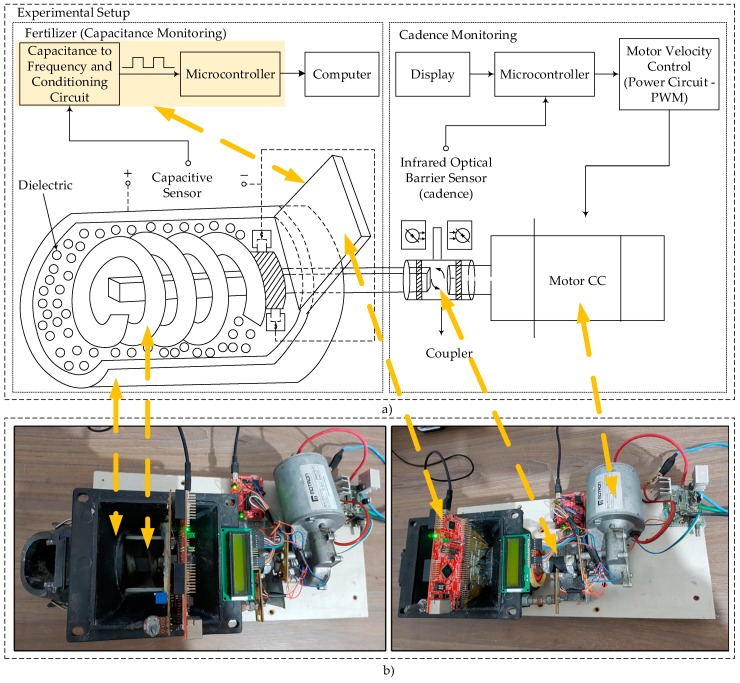
(**a**) Diagram of the developed prototype scheme. (**b**) Photos in two different profiles for better visualization of the parts of the prototype used in bench tests.

Figure 4a presents the block diagram of the experimentation of the prototype developed: on one side, the prototype is shown with the presence of the mobile helical capacitive sensor, signal conditioning circuits, data pre-processing and digitalization; and on the other, a CC engine and its coupling to the sensor, along with the cadence sensor, a microcontroller for the speed control and the engine triggering power circuit. Figure 4b illustrates an assembled prototype of the same type as that was used in the experimental tests.

### 2.4. Experimental Methodology

The sensor response analysis employed eight different fertilizer formulations resulting from the mixture of simple fertilizer granules, known as NPK formulations, which were 00-00-60, 46-00-00, 02-24-12, 02-28-20, 02-20-10, 00-25-25, 04-24-12 and 06-21-12. Each fertilizer formulation was submitted to moisture, granulometry, real and apparent density analyses. 

Each sample moisture content was obtained using the thermogravimetric method, through an infrared halogen analyzer, where the sample initial weight was stored and then exposed to a halogen radiator for drying. Integrated scales stored the sample weight loss, and after about three minutes, the moist mass weight difference in relation to the dry mass was interpreted as moisture content.

For the granulometric analysis, 270 g of each fertilizer formulation was separated and a set of sieves was selected according to the Tyler series. The sieves were fitted to each other over an automatic agitator, and the sieve with the largest aperture was placed on top of the others. Each sample was poured on the sieve set and agitated for 5 min. After the agitation process, the material remaining in each sieve was weighed and the values converted into percentages for due comparison between the sieves.

The real density of each fertilizer formulation was obtained using a 100 mL test tube and precision scales. The test tube was filled with a sample of the fertilizer formulation and its mass was calculated, thus obtaining the real density of each fertilizer formulation.

For the calculation of apparent density, the sample total volume was taken into consideration, including the empty spaces. Soybean oil with a 0.891 g/cm³ density was added to the test tube containing the fertilizer, so that the empty spaces between the granules were filled. The volume of oil needed to fill up the empty spaces was deducted from the initial volume, thus obtaining the apparent density of each fertilizer formulation.

After the physicochemical analysis was carried out, the static and dynamic tests were performed, with the main objective of verifying the presence, absence or variations in the fertilizer distribution due to obstruction. Another two secondary analyses were also carried out: the former refers to the possibility of verifying the formulation of the fertilizer present in the dosing mechanism due to the different characteristics of each formulation, while the latter refers to the influence of moisture content in the fertilizer dielectric permittivity and consequent sensor capacitance.

For each experiment carried out, four samples of capacitance per second were collected, for 60 s, resulting in 240 capacitance samples. These 240 samples were selected using a digital filter which provides a cumulative mobile mean of input data and returns a capacitance sample. The room temperature and the relative moisture at the time of the experiments varied from 18 to 25 °C and from 40 to 60%, respectively.

### 2.5. Experimental Protocol

The protocol used to carry out the experiments is described below:

- **Experiment 1**: Analysis of the presence and absence of fertilizer

**Static analysis in the absence of the fertilizer:** The helical electrode was positioned and the data capture and processing started and were carried out for 60 s.

**Dynamic analysis in the absence of fertilizer:** The engine rotation was adjusted so that it corresponded to the desired distribution speed; next, data capture and processing started and were carried out for 60 s.

**Static analysis in the presence of fertilizer:** The fertilizer was added to the dosing mechanism, and the engine was started for a certain time so that the fertilizer could flow and fill up the inside of the dosing mechanism up to the point of being released to the soil; at this point, the engine was turned off. The helical electrode stopping position was the same for all experiments. Next, data were captured and processed for 60 s.

**Dynamic analysis in the presence of fertilizer:** The fertilizer was added to the dosing mechanism and the engine was started in the desired rotation, and the fertilizer was released. Data were captured and processed for 60 s from the moment the fertilizer iwasspread on the soil, preventing differences in the sensor response due to the spaces that might not have been filled by the fertilizer inside the dosing mechanism.

**Fertilizer distribution process full cycle analysis:** The engine was started, adjusting the dosing mechanism axis rotation speed. Data capture and processing were carried out for 60 s, starting with the empty dosing mechanism, which after 15 s received a constant flow of fertilizer in its reservoir and remained full for approximately 30 s. Next, the fertilizer supply to the reservoir was interrupted and the mechanism was allowed to release all the fertilizer.

- **Experiment 2**: Distribution variation analyses

**Dynamic analysis in the presence of fertilizer and one obstruction:** The fertilizer was added to the reservoir and the engine was adjusted with the desired speed. Data capture was carried out for 60 s when the fertilizer was about to be released to the soil. After about 15 s, the reservoir was obstructed, simulating fertilizer aggregation. After approximately 5 s from the start of the obstruction, the fertilizer flow started to be reduced. The obstruction was kept for around 20 s, and then removed, letting the dosing mechanism release all the fertilizer.

- **Experiment 3:** Identification of different dielectrics

The procedures adopted for the experiment are the same as those adopted for the dynamic analysis in the presence of fertilizer. After data collection and processing, a mobile mean of the 240 capacitance value samples was carried out, aiming at verifying the capacitance band in which each fertilizer formulation would work. Next, the software Excel and Past were used for the statistical analysis (ANOVA) and the Tukey test, to verify whether each fertilizer formulation capacitance means differed statistically.

- **Experiment 4:** Moisture influence in the fertilizer dielectric permittivity

A sprayer was used to add water particles to eight samples of two fertilizer formulations. Four samples corresponded to the formulation 02-20-10 and the other four to the formulation 02-28-20. Next, the moisture content of the samples was verified and the sensor response was analyzed with the addition of fertilizer to the sensor. To reduce errors related to mechanical backlash, granule crushing or other variables inherent in the process and effectively verify the moisture influence, the experiment was carried out in static mode and without moving the helical electrode. Therefore, the inside of the dosing mechanism was not totally filled, presenting capacitance values different from those found in the remaining experiments. Likewise, after each test, the fertilizer was removed manually without rotating the axis and remaining in the same position as in the other experiments.

Except for experiment 4, the electrodes that made up the sensor were removed and cleaned after each experiment. This procedure might result in small capacitance differences due to the sensor assembling process and the mechanical characteristics of the dosing mechanism, such as axis decentralization and backlash.

## 3. Results and Discussion

### 3.1. Identification of Fertilizer Presence and Absence

The first results are shown in Table 1, which presents the capacitance mean of all formulations and the capacitance difference in the absence and presence of fertilizer highlighting the sensor ability to identify these situations.

The capacitance values show an approximately 1% difference between the experiments carried out in static and dynamic modes due to the movement of the helical electrode, prototype mechanical backlash and granulometric variations between samples. 

**Figure 5:**
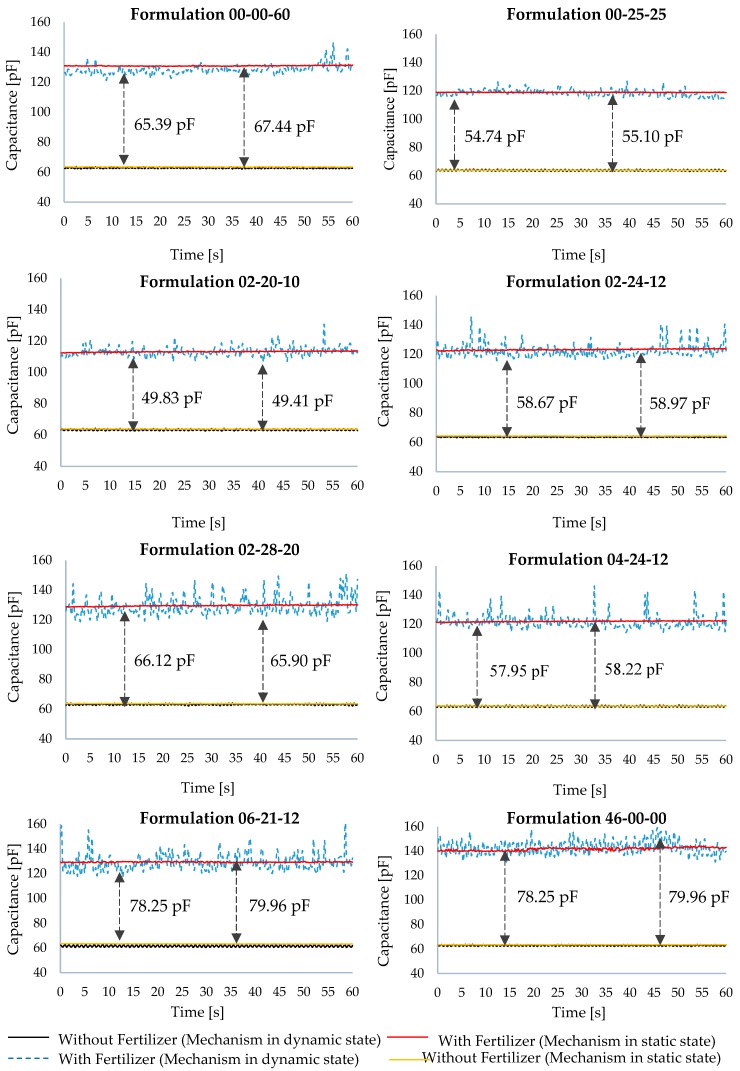
Graphs of the sensor response to the fertilizer presence and absence.

Figure 5 presents the graphs of each fertilizer formulation, expressing graphically the presence or absence of fertilizer. Each graph presents four curves, which represent the capacitance values in static and dynamic modes. The two lower curves (yellow and black) present the mechanism capacitance values in the absence of fertilizer, while the upper curves (blue and red) represent the capacitance values in the presence of fertilizers. The values referring to the difference between fertilizer presence or absence presented in the graphs are the results of the mean of the 240 samples collected in static and dynamic modes.

Empirical observations carried out during the experiments revealed that the greatest oscillations occurred due to the granules that got stuck between the electrodes; they were crushed, causing capacitance peaks. The granulometric analysis presented in Table 2, confirms this information, indicating that the formulation 06-21-12 presented the highest number of particles larger than 4mm, thus favoring the crushing of the granules due to the distance between the electrodes, which was 3mm.

Table 3 presents the basic statistics of the data collected referring to the identification of the fertilizer presence or absence, showing the coefficient of variation (VC), maximum capacitance (MAX), minimum capacitance (MIN) and mean capacitance (MEA). 

Analyzing the data dispersion measurements presented in Table 3, where VC expresses the extension of the data variability in relation to the population mean in percentage, and the SDV refers to the error in relation to the population area, the highest VC and SDV were presented in dynamic mode with the presence of fertilizers in the formulation 06-21-12 with values of 5.53% and 7.14 pF, respectively; greater data dispersion was seen in this formulation around the arithmetic mean. However, all the formulations presented a VC below 10%, indicating data homogeneity and low dispersion in relation to the arithmetic mean.

**Figure 6:**
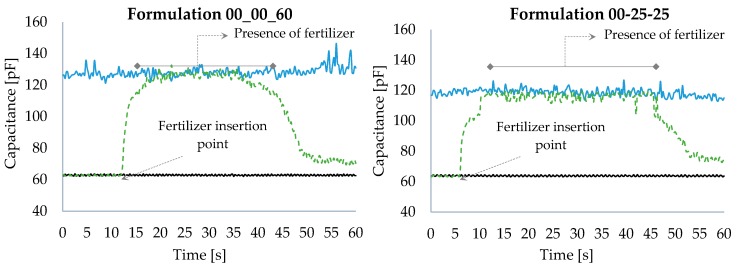

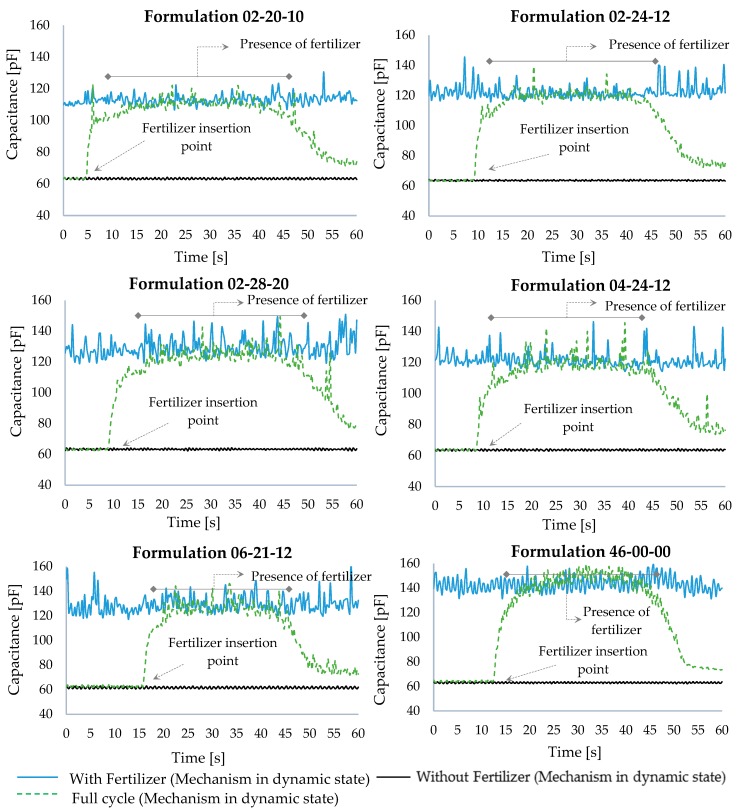
Graphs of the sensor response simulating the fertilizer distribution full cycle.

Figure 6 illustrates the sensor response simulating the full fertilizer distribution cycle. The upper and lower curves (blue and black) are the reference for the fertilizer presence and absence, respectively, both in dynamic mode, while the broken-line curve (green) illustrates the fertilizer distribution full cycle. 

When analyzing the curve corresponding to the fertilizer distribution full cycle, the capacitance values presented are seen to be very close to the reference values, corresponding to the mechanism in the absence of fertilizer. After a certain period, the fertilizer is added to the dosing mechanism. At that point, the sensor increases its capacitance presenting values corresponding to the dosing mechanism in the presence of fertilizer. The transition time between the presence and absence of fertilizer is observed to be relatively short, demonstrating that the sensor developed a short response time to presence. After fertilizer addition, the sensor remains at capacitance levels corresponding to the mechanism in the presence of fertilizer, up to the moment in which the fertilizer in the reservoir is totally released; at that point, the capacitance values go back to the values corresponding to the mechanism in the absence of fertilizer.

However, a difference is noticed in the capacitance at the end of the process in relation to that considered ideal (in the absence of fertilizer and dynamic mode). This difference refers to the accumulation of fertilizer that remains inside the sensor, due to its characteristic of being an overflow dosing mechanism; that is, it presents a regulator at the level of the output aperture of the mechanism that accumulates the product, forcing it to overflow to reach the soil. This fertilizer overflow enables the reduction of distribution variations caused by the “pulses” generated at the end of each helical cycle. 

### 3.2. Variations in Fertilizer Distribution

**Figure 7:**
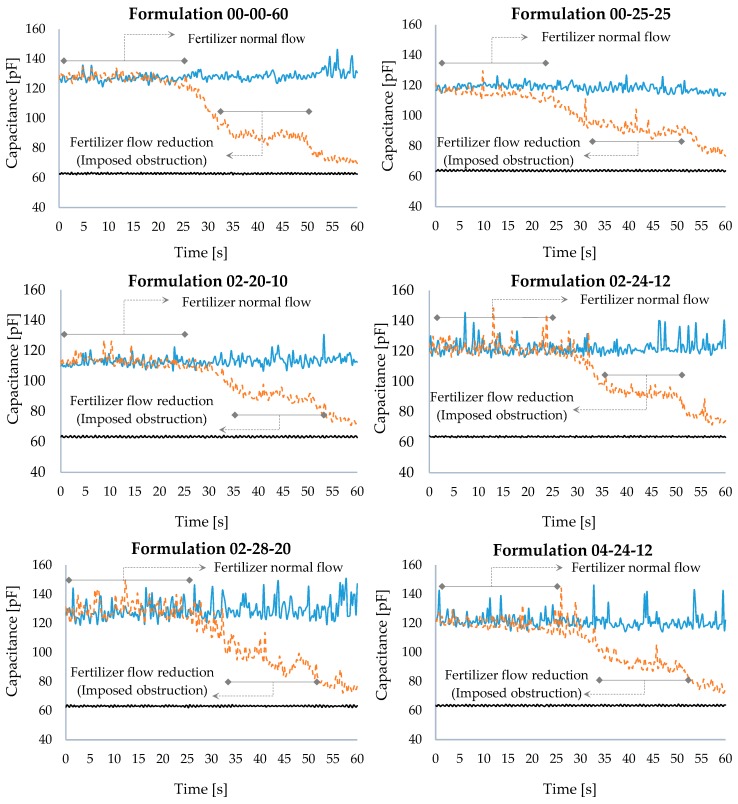

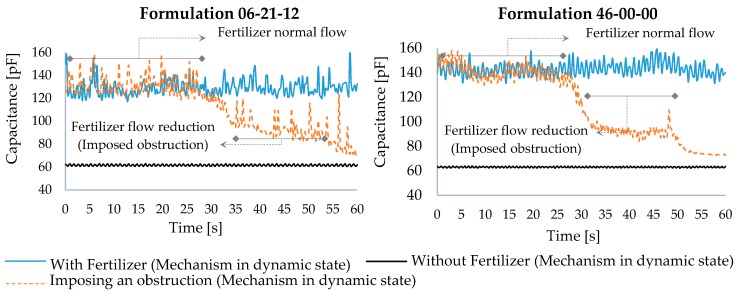
Graphs of the sensor response to the imposition of an obstruction.

Figure 7 illustrates the results obtained by the imposition of an obstruction at the fertilizer input aperture in the dosing mechanism, provoking a reduction in the pre-set normal flow. The graphs show three curves: the upper and lower curves (black and blue) refer to the absence and presence of fertilizers, respectively. The central broken-line curve (orange) presents the capacitance variation due to the reduction in the fertilizer flow due to the obstruction created.

Initially, when fertilizer was added, the sensor response was noticed to present values close to those obtained when the experiments were carried out with the dosing mechanism in the presence of fertilizers in the dynamic mode. From the imposition of the obstruction, at point t = 25 s, the sensor capacitance was observed to reduce due to the decrease in the fertilizer flow. Taking the formulation 00-00-60 with both dosing mechanisms as an example, the capacitance value was seen to reduce by approximately 58% around points t = 30 and t = 50, keeping values close to 90 pF and presenting oscillations resulting from the lack of control of the amount of fertilizer retained by the obstruction. From point t = 50 on, the capacitance values tended to get closer to those referring to the dosing mechanism in the absence of fertilizer, due to the total release of the fertilizer contained in the reservoir by the mechanism.

This analysis enabled the identification of fertilizer distribution variations, resulting from the fertilizer’s normal flow reduction, either due to obstructions or other variations related to the fertilizer characteristics, such as lack of fluidity, which causes a reduction in the pre-set flow. Thus, the sensor is characterized as a new tool to help farmers, enabling homogeneous and more profitable fertilization.

### 3.3. Fertilizer Formulation Identification Test

**Figure 8:**
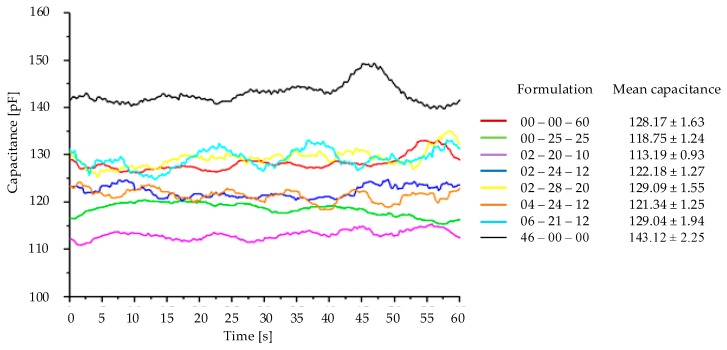
Mobile mean of 20 samples with the overflow dosing mechanism.

Figure 8 presents the results referring to the analysis to identify the fertilizer formulation contained in the dosing mechanism. Each formulation is illustrated by a curve in the graph; the mean capacitance is also presented along with the standard deviation of each formulation, resulting from the mobile mean of 20 samples. 

The formulations 00-00-60, 02-28-20 and 06-21-12 were seen to present mean capacitances which were very close one to each other; however, when analyzing the sample moisture, they were seen to be significantly different, influencing the capacitance presented by the sensor. The same occurred with the formulations 02-24-12 and 04-24-12; however, these were only different one from another due to the amount of nitrogen in their formulation, which might explain the closer capacitance values of these two formulations.

Aiming at identifying statistically whether there was a significant difference between the fertilizer formulation means, the statistical analysis ANOVA was carried out followed by the Tukey test, considering a 5% significance level. The ANOVA results presented value−p < 0.05 and $F>F_{Critical}$ confirming statistically that there is evidence that at least one of the capacitance means of the fertilizer formulations was different from the others. The Tukey test showed that only formulations 02-28-20 and 06-21-12 did not differ statistically one from another. All the other fertilizer formulations presented statistically different capacitance values, considering a 5% significance level. Table 4 presents the mean capacitance of the fertilizer formulations considering the mobile mean carried out, highlighting the formulations that are statistically different, along with the standard deviation, variation coefficient and their respective moisture content.

Taking the formulations that present a similar moisture content as a basis of comparison, which reduces the error imposed by moisture, such as the formulations 00-25-25 and 02-28-20 with 2.52% and 2.48% moisture content, the sensor response was seen to act in distinct capacitance bands 118.75 pF and 129.09 pF. The same occurred in formulations 02-20-10 and 02-24-12 with 1.66% and 1.7% moisture content, producing distinct responses, 113.19 pF and 122.18 pF. Different capacitance bands were also reported in [25], which tested urea, superphosphate and potassium sulphate in a capacitive sensor coupled to the fertilizer output duct.

Mechanical problems still must be eliminated, reducing most of the capacitance variations caused by the crushing or the helical electrode displacement, and a constant monitoring of the moisture content is also needed for possible corrections in order to confirm the sensor’s potential to identify fertilizer formulations. However, considering the limitations of this project’s development, the sensor has already shown great potential to identify changes in the fertilizer characteristics regarding distribution, indicating the possibility of formulation alteration during the distribution.

### 3.4. Moisture Influence in the Fertilizer Dielectric Permittivity

**Figure 9:**
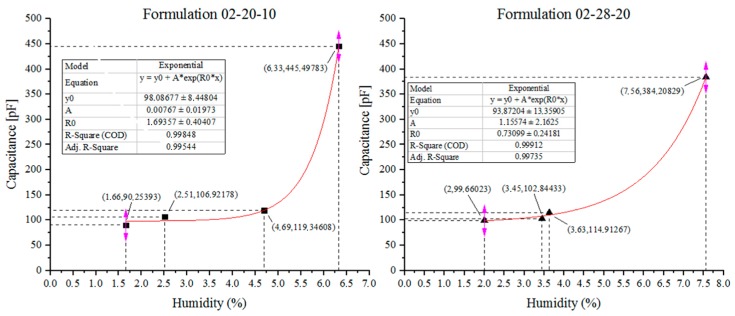
Sensor response to different moisture contents in the formulations 02-20-10 and 02-28-20.

Figure 9 shows the sensor response to the different levels of moisture in the formulations 02-20-10 and 02-28-20. In the formulation 02-20-10, the moisture contents tested were 1.66%, 2.51%, 4.69% and 6.33%, presenting 90.25 pF, 103.92 pF, 119.35 pF and 445.50 pF capacitance variation, respectively. In the formulation 02-28-20, the moisture contents tested were 2%, 3.45%, 3.63% and 7.56%, resulting in 99.66 pF, 102.84 pF, 114.91 pF, and 384.21 pF, respectively.

The moisture content influenced the dielectric permittivity of the fertilizers, resulting in an alteration of the capacitive sensor capacitance. Both tests showed that the capacitance variation in relation to moisture content was relatively low up to around 4%, while the most significant alterations occurred when the moisture content was around 6.33% and 7.56%, with an increase in capacitance of around 493.60% and 385.51% when compared to the 1.66% and 2% initial moisture. Considering that the fertilizers leave the factory with a moisture below 1% [25], these values would not cause significant capacitance alteration. 

Due to their hygroscopic characteristics, the fertilizers present a relative critical moisture, which can be defined as the moisture of the atmosphere above which the fertilizer spontaneously absorbs moisture, which might occur due to improper transportation or storage [37]. Therefore, monitoring moisture content during distribution is important to obtain measurements that effectively represent the values measured.

## 5. Conclusions

The developed sensor was based on a commercial fertilizer dosing mechanism, promoting the least physical alteration possible. Therefore, based on empirical results and experimental analyses, its efficacy was proved, and it might be considered a new tool to help farmers to detect both the presence or absence of fertilizers and variations in the distribution caused by obstruction or any other factor that might reduce the pre-set fertilizer flow.

The sensor identified the absence or presence of fertilizers, regardless of the formulation tested in 100% of the experiments carried out. All the data collected presented a VC below 10%, suggesting homogeneity and a low dispersion of data around the arithmetic mean.

In all experiments carried out to identify variation in the distribution due to the imposition of an obstruction, the sensor identified reduction in the fertilizer output flow and, consequently, reduced its capacitance.

The sensor demonstrated its potential to identify the fertilizer formulation contained in the dosing mechanism. Only formulations 02-28-20 and 06-21-12 did not differ statistically according to the ANOVA analysis and the Tukey test at a 5% significance level. 

The sensor also presented significant capacitance variations when specific experiments were carried out by inserting different moisture contents in the formulations 02-20-10 and 02-28-20. Thus, further studies are necessary to verify the need for the introduction of sensors to monitor and correct alterations caused by other factors included in the process, such as moisture.

Although the dosing mechanism still presents mechanical faults such as axis backlash, resulting in errors that must be adjusted to improve the stability of the sensor response, the results obtained already confirm its ability to identify the presence or absence and variations of fertilizer in the distribution of solid granular fertilizers.

## 6. Patents

Privilege of innovation, title: “Mobile helical capacitive sensor device for analysis of fertilizer distribution”, Brazil, 2018. Institution of registration: National Institute of Industrial Property (INPI). Register number: BR1020180128329. Date deposit: 21 June 2018.

## Figures and Tables

**Table 1:** Capacitance difference in the absence and in the presence of the dielectric.

Formulation	Static			Dynamic		
	Without Fertilizer	With Fertilizer	Difference	Without Fertilizer	With Fertilizer	Difference
	(pF)	(pF)	(pF)	(pF)	(pF)	(pF)
00-00-60	63.45	130.89	67.44	62.88	128.27	65.39
00-25-25	63.86	118.96	55.10	63.81	118.55	54.74
02-20-10	63.73	113.13	49.41	63.30	113.13	49.83
02-24-12	64.22	123.19	58.97	63.63	122.28	58.65
02-28-20	63.66	129.55	65.90	63.20	129.33	66.12
04-24-12	63.67	121.89	58.22	63.51	121.45	57.95
06-21-12	63.36	129.33	65.97	61.62	129.20	67.58
46-00-00	63.54	141.79	78.25	63.02	142.98	79.96

**Table 2:** Granulometric analysis results.

Formulation	P4	P5	P7	P8	P10	P14	P18	P35
	4.76 mm	4 mm	2.83 mm	2.38 mm	2 mm	1.41 mm	1 mm	0.5 mm
	P.P (%)	P.P (%)	P.P (%)	P.P (%)	P.P (%)	P.P (%)	P.P (%)	P.P (%)
00-00-60	100	99.64	59.25	24.01	0.64	0.03	0	0
00-25-25	100	99.34	51.06	18.48	0.92	0.02	0	0
02-20-10	100	98.8	64.12	28.35	1.39	0.03	0.01	0.01
02-24-12	100	98.7	70.21	34.58	2.5	0.07	0.02	0.01
02-28-20	100	99.41	75.7	44.8	5.08	0.21	0.03	0
04-24-12	100	98.45	67.94	29.2	1.08	0.05	0.02	0
06-21-12	100	97.77	63.5	22.55	0.93	0	0	0
46-00-00	100	100	86.7	39.63	0.23	0.02	0.01	0

P.P—Passing particle.

**Table 3:** Basic descriptive statistics of the sensor response with the overflow dosing mechanism.

	Static without Fertilizer	Dynamic without Fertilizer	Static with Fertilizer	Dynamic with Fertilizer
**00-00-60**				
SDV	0.14	0.43	0.20	3.09
VC(%)	0.22	0.68	0.15	2.41
MAX	63.82	63.71	131.47	146.44
MIN	63.07	62.03	130.50	121.22
MEA	63.45	62.88	130.89	128.27
**00-25-25**				
SDV	0.06	0.43	0.09	2.42
VC(%)	0.09	0.68	0.08	2.05
MAX	64.02	64.59	119.20	126.82
MIN	63.70	62.97	118.71	112.90
MEA	63.86	63.81	118.96	118.55
**02-20-10**				
SDV	0.06	0.47	0.31	3.05
VC(%)	0.10	0.74	0.28	2.69
MAX	63.89	64.22	113.70	130.67
MIN	63.55	62.54	112.17	106.57
MEA	63.73	63.30	113.13	113.13
**02-24-12**				
SDV	0.07	0.34	0.42	4.69
VC(%)	0.11	0.53	0.34	3.83
MAX	64.37	64.33	123.95	145.52
MIN	63.99	63.00	122.05	115.74
MEA	64.22	63.63	123.19	122.28
**02-28-20**				
SDV	0.09	0.44	0.42	6.31
VC(%)	0.14	0.70	0.32	4.88
MAX	63.99	64.15	130.37	150.25
MIN	63.43	62.09	128.60	118.86
MEA	63.66	63.20	129.55	129.33
**04-24-12**				
SDV	0.08	0.44	0.29	5.08
VC(%)	0.13	0.70	0.24	4.18
MAX	63.92	64.40	122.47	146.26
MIN	63.45	62.72	121.18	114.20
MEA	63.67	63.51	121.89	121.45
**06-21-12**				
SDV	0.12	0.75	0.30	7.14
VC(%)	0.19	1.21	0.23	5.53
MAX	63.64	62.95	129.80	164.32
MIN	63.04	60.50	128.80	117.04
MEA	63.36	61.62	129.33	129.20
**46-00-00**				
SDV	0.06	0.50	1.21	5.78
VC(%)	0.10	0.79	0.86	4.04
MAX	63.71	63.87	144.33	158.90
MIN	63.32	62.11	139.42	131.21
MEA	63.54	63.02	141.79	142.98

SDV—standard deviation; VC—variation coefficient; MAX—maximum; MIN—minimum; MEA—mean.

**Table 4:** Mobile mean of each fertilizer formulation, standard deviation and respective moisture content.

Formulation	Mean Capacitance (pF)	VC (%)	Standard Deviation (pF)	Moisture (%)
00-00-60	128.17 *	1.27	1.63	0.25
00-25-25	118.75 *	1.04	1.24	2.52
02-20-10	113.19 *	0.82	0.93	1.66
02-24-12	122.18 *	1.04	1.27	1.70
02-28-20	129.09 **	1.20	1.55	2.48
04-24-12	121.34 *	1.03	1.25	1.45
06-21-12	129.04 **	1.50	1.94	1.17
46-00-00	143.12 *	1.57	2.25	0.43

(*) Differ statistically (*p* < 0.05), (**) Does not differ statistically (*p* < 0.05).
